# Editorial: Anthropogens, lifestyle and pathophysiology of chronic diseases: From mutual interplay to translational research and personalized medicine

**DOI:** 10.3389/fmed.2022.1120066

**Published:** 2022-12-23

**Authors:** Daniela Calina, Anca Oana Docea, Antonio F. Hernández, Aristidis M. Tsatsakis, Ileana Mardare

**Affiliations:** ^1^Department of Clinical Pharmacy, University of Medicine and Pharmacy of Craiova, Craiova, Romania; ^2^Department of Toxicology, University of Medicine and Pharmacy of Craiova, Craiova, Romania; ^3^Department of Legal Medicine and Toxicology, University of Granada, Granada, Spain; ^4^Health Research Institute of Granada (ibs.GRANADA), Granada, Spain; ^5^Consortium for Biomedical Research in Epidemiology and Public Health (CIBERESP), Madrid, Spain; ^6^Department of Forensic Sciences and Toxicology, Faculty of Medicine, University of Crete, Heraklion, Greece; ^7^Department of Public Health and Management, “Carol Davila” University of Medicine and Pharmacy of Bucharest, Bucharest, Romania

**Keywords:** anthropogens, lifestyle, pathophysiology, chronic diseases, translational research, personalized medicine

Modern man-made environments, lifestyles, and behaviors (also known as anthropogens) have the ability to induce long-term low-level systemic inflammation (meta-inflammation) that may activate the inflammatory-oxidative cascade ultimately resulting in the development and progression of a wide range of chronic diseases (CD). These include cardiovascular diseases, metabolic disorders, cancer, among others, with high morbidity and mortality rates worldwide (1–3).

The interaction of magnitude and duration of exposure to anthropogens and chemicals with individuals' genetic background and/or epigenetic changes may represent the early stages of the pathogenesis of the chronic cardio-metabolic diseases mentioned earlier (4).

For effective disease management strategies, health professionals should focus on identifying all the potential risk factors associated with meta-inflammation and on tackling these through personalized health care services.

The aim of this Research Topic was to bring together the newest research results in the field of anthropogens, lifestyle (with a focus on nutrition), the pathophysiology of chronic diseases, and potential interventions for the prevention and management of chronic diseases, particularly at individual level.

Considering the high diversity of anthropogenic factors, the contributors covered a wide range of Research Topics, focusing on environmental risk factors, lifestyle, potential interventions, and outcomes measurement, most of them providing original research results (8/9 articles).

In a population-based cohort study, He et al. analyzed pre- and perinatal risk factors, and found that even a normal Apgar score of 7–9 may predict a suboptimal brain development and higher risk of poor short and long-term outcomes, such as mental disorders, organic disorders, and neurodevelopmental disorders. The results of this study, performed with data from Danish national registries, suggested that suboptimal Apgar scores < 7–9 should be also considered an alarming risk factor for subsequent mental disorders and for designing targeted public health strategies.

Giambò et al. reviewed the role played by the gut microbiota regarding the metabolism and protection mechanisms against environmental chemicals (i.e., pesticides, metals, and microplastics) to tailor personalized preventive strategies. The human gut microbiota may interact with environmental pollutants, such as pesticides, metals, and microplastics, which are incriminated as risk factors for cancer, obesity, diabetes, immune disorders, and etc. GM composition analysis, together with the determination of well-known biomarkers, could be used as a screening tool for assessing the long-time exposure to pollutants and shaping specific preventive interventions.

Eguiguren-Jiménez et al. performed a retrospective cross-sectional study focused on the risk factors associated with chronic kidney diseases (CKD) in non-institutionalized adults in Quito, Ecuador's capital city. Based on data registered between 2019 and 2021 on a randomly selected sample of 1,701 adults, out of whom 813 met the inclusion criteria, indicated a CKD prevalence of 7.2%, with about 45% in diseases stages 2–4. Systolic blood pressure, sex and residence area (probably linked to environmental factors) were the main risk factors significantly associated with estimated glomerular filtration rate (eGFR) as a proxy for CKD.

Xenos et al. analyzed the impact of Vitamin D supplements on the outcome of weight loss diets in Caucasian population, through a randomized double-blind placebo-controlled clinical study on vitamin D3 supplementation together with personalized weight-loss diet on obesity markers in overweight and obese individuals with vitamin D deficiency or insufficiency. The study found that 3,000 IU vitamin D3 oral spay decreased obesity markers, with the response being influenced by the vitamin D receptor (VDR) and adrenergic receptors (ADRs) genetic polymorphism. Edo et al. also dedicated their research to the role of dietary factors, investigating the potential association between the nutrients intake and the retinal vessels caliber in a cross-sectional survey performed in Japanese descendants living in Los Angeles. The study found a significant inverse association between vitamins A, C, and potassium intake and retinal venular caliber, indicative of a beneficial effect of these nutrients on the retinal microvascular profile and a healthier retinal micro-circulation.

Li et al. performed a cross-sectional study to examine the association between a lifestyle component—leisure time physical activity and multi-morbidity (17 chronic diseases, representing the most common non-communicable diseases among Chinese population). The study was based on 6,084 participants from a large-scale, multi-ethnic cohort in China. Results showed significant associations between low level of physical activity and chronic diseases, suggesting that health interventions should be specifically designed.

Two articles were dedicated to potential interventions addressing risk factors or symptoms of dyslipemia and chronic prostatitis. Lee and Lee presented the results of a randomized double-blind placebo-controlled trial, assessing the lipid-lowering effects of *Ulmus macrocarpa Hance extract* (UME) in adults with untreated high low-density lipoprotein cholesterol concentration. After 12 weeks, the results indicated that the lipid profile of patients treated with UME improved significantly as compared with the placebo group. Searching for an effective and safe solution for chronic prostatitis (CP)/chronic pelvic pain syndrome (CPPS), the most common chronic prostatitis in men, Wu et al. investigated the analgesic mechanism of electroacupuncture (EA), the combination between traditional acupuncture and modern electrical stimulation, in rats with CP/CPPS. Results showed that the analgesic mechanism of EA may be related to the cAMP-PKA-TRPV1/PLC-PKC-TRPV1 signaling pathway, by normalizing the altered gene expression.

Finally, Pan et al. investigated the model for end-stage liver disease (MELD) score and the changes on the dynamic changes in circulating lymphocyte subsets in Chinese patients undergoing liver transplantation. The study showed that patients with either the low MELD scores or the long-term follow-up period are in a relatively good immune condition and may receive higher doses of immunosuppressants, to prevent the potential complications.

Overall, the studies included in this Research Topic highlight how lifestyle, nutritional and environmental interventions, apart from specific pharmacologic treatments, can influence the prognosis of chronic diseases and contribute to identify new mechanisms involved in their pathogenesis.

## Author contributions

All authors listed have made a substantial, direct, and intellectual contribution to the work and approved it for publication.
